# The Emerging Role of Platelets in the Formation of the Micrometastatic Niche: Current Evidence and Future Perspectives

**DOI:** 10.3389/fonc.2020.00374

**Published:** 2020-03-18

**Authors:** Stavros Gkolfinopoulos, Robin L. Jones, Anastasia Constantinidou

**Affiliations:** ^1^BOC Oncology Center, Nicosia, Cyprus; ^2^The Royal Marsden Hospital NHS Foundation Trust and Institute of Cancer Research, London, United Kingdom; ^3^Medical School, University of Cyprus, Nicosia, Cyprus; ^4^Cyprus Cancer Research Institute, Nicosia, Cyprus

**Keywords:** platelets, microenvironment, cancer, metastatic, niche, antiplatelet

## Abstract

Accumulating evidence suggests that platelets play a key role in cancer metastatic dissemination through their multilevel interaction with tumor cells. Most crucial is the contribution of platelets to the formation and expansion of the early metastatic niche, a protective microenvironment that nurtures the first metastatic cells and is necessary for the establishment of overt metastatic disease. A multitude of mechanisms have been proposed toward this effect. The current review examines the implication of platelets in the three most well-studied mechanisms: (a) the initial preparation of the metastatic microenvironment by the formation of the extracellular matrix (ECM) and the recruitment of granulocytes, (b) the creation of the neovasculature (important for providing the developing tumor with oxygen and nutrients and clearing away the metabolic waste), and (c) the evasion of the immune response by the creation of an immune-suppressive environment around the developing metastases. Finally, the review provides current perspectives on the potential clinical relevance of platelets in cancer progression and their consequent role in cancer therapeutics.

## Introduction

Derived from the megakaryocytes, platelets are small fragments of circulating cytoplasm with a key role in primary hemostasis. Increasing evidence in recent years supports their critical role in cancer progression and particularly in metastatic dissemination through their multilevel interaction with tumor cells.

The formation of the micrometastatic niche is depended upon the arrival of circulating tumor cells (CTCs) to sites distant to the primary site. Preclinical evidence now suggests that platelets have a particular role in the formation of the “early metastatic niche” (1, 2) based on the hypothesis that platelet-derived signals, in addition to signals derived from the tumor itself, are responsible for the recruitment of granulocytes in the early metastatic sites, where cancer cells begin to accumulate (3). The recruitment of a variety of host-derived cells, that will eventually form the tumor stroma, is mediated by the chemokines CXCL5 and CXCL7, which are secreted by the platelets that become activated after interacting with the tumor cells. Blockade of the CXCR2, which is the CXCL5/7 receptor, may result in significant reduction of metastatic spread and cancer progression (4).

During their journey through the circulation, CTCs adhere to circulating platelets by adhesion molecules expressed on their surface, like the tissue factor and P-select in ligands (5). In this way, CTCs are engulfed in a protective shield of platelets that not only prevent their lysis from natural killer (NK) cells, but also facilitate their adhesion to the endothelium and their subsequent extravasation (6). Additionally, platelets increase their metastatic potential by triggering the TGFb-1 and NF-kB pathways that are responsible for the epithelial-mesenchymal transition (7).

After their extravasation and the loss of their protective coating, tumor cells are in the danger of undergoing apoptosis through a process called anoikis (detachment-induced apoptosis). This results from the lack of a protective surrounding environment, and isolated cancer cells are subject to this fate, unless they manage to discover a new home in the site where they metastasize (8). This new home for the errant tumor cells is known as micrometastatic niche, and platelets, once again, constitute the major driving force for its creation (9).

The current review presents available evidence on the implication of platelets in the creation of the metastatic niche through the formation of the extracellular matrix, the building of the neovasculature and the establishment of the immune response. The future potential application of this knowledge in the clinical setting is also discussed here.

## The Creation of the Metastatic Niche

**Figure 1:**
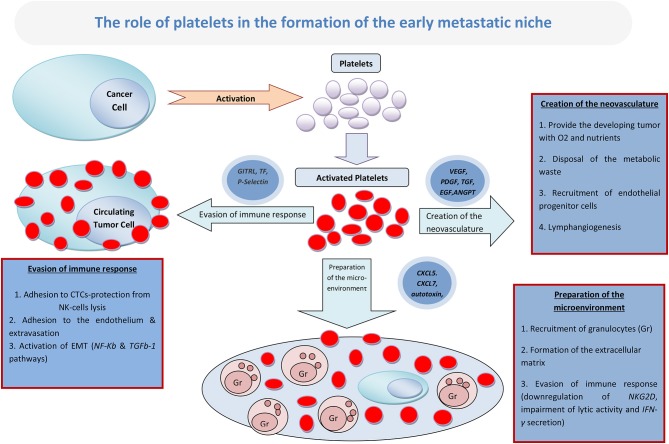
The role of platelets in the formation of the early metastatic niche.

Although the various processes leading to the creation of the metastatic niche may be overlapping, they can be divided in three major phases: the initial preparation of the metastatic microenvironment by the formation of the extracellular matrix (ECM) and the recruitment of granulocytes; the creation of the neovasculature, which is important for providing the developing tumor with oxygen and nutrients, as well as for clearing away the metabolic waste; and, lastly, the evasion of the immune response by the creation of an immune-suppressive environment around the developing metastasis (Figure 1).

### Preparing the Metastatic Microenvironment

There is increasing evidence to support the idea that platelets initiate the shaping of the metastatic microenvironment in the context of early metastatic niche. This has been shown in a lung cancer murine model, where tumor-aggregated platelets have guided the creation of metastatic sites by the production of CXCL-5 and CXCL-7 cytokines that attract granulocytes (4). Furthermore, platelets may be responsible for the development of osteoblastic and osteolytic bone metastasis, as has been demonstrated in a study by Kerr et al., where tumor-induced bone formation was impaired following platelet depletion (10). Also, studies by Peyruchaud et al. have shown that tumor-activated platelets may guide bone colonization by breast cancer cells and create lytic bone metastases (11, 12). The proposed mechanism involves the secretion of autotaxin by activated platelets, which subsequently binds to tumor cell integrin ανβ3, promoting the conversion of lysophosphatidylcholine (LPC) to lysophosphatidic acid (LPA). Subsequently, the autocrine-acting LPA activates tumor LPA receptors, inducing the secretion of cytokines that stimulate the osteoclast-mediated bone destruction (12).

The early metastatic niche is formed mostly by ECM, by platelets and by the granulocytes that, as described above, are recruited on site by the activated platelets (13). In a study on a lung cancer model, the knockout of platelet ADP receptor (P2Y12) led to decreased lung fibronectin, which is a major component of ECM. Fibronectin is increased in the connective tissue of a pre-metastatic organ, and it is one of the most vital components of the acellular matrix of the metastatic niche. In this model, this platelet-induced downregulation of fibronectin resulted in decreased rate of metastases (14).

Conclusively, it appears that platelets can play a vital role in the very early formation of both the cellular and the acellular elements of the early metastatic microenvironment. The formation of a supportive structure consisting of ECM and host-derived cells is a prerequisite for the successful establishment of metastases.

### Promoting the Creation of Neovasculature

The formation of new blood vessels from pre–existing ones in cancer is called neoangiogenesis (15). This process is particularly important for tumors sized >2 mm; consequently its successful completion is crucial for the development of the early metastatic site (16). Cancer growth rate is mainly influenced, and by extent, limited, by the formation of new blood vessels, which is primarily guided by platelets (17, 18). Indeed, platelets are the main transporters of proangiogenic factors, such as the vascular-endothelial growth factor (VEFG) (19). The pivotal role of platelets begins at the very early steps of vasculogenesis and continues until the advanced stages (20). Platelets need to be activated by tumor cells in order to fulfill this role, and this tumor cell-induced platelet activation (TCIPA) is characterized by platelet aggregation, adhesion, and an increase in both platelet numbers and platelet-derived pro-angiogenic factors (21).

The mechanisms through which platelets contribute in neoangiogenesis have been well-characterized (20). Initially, platelets become activated after coming into contact with subendothelial structures, such as collagen, in places with abnormal blood flow, such as the metastatic sites. The subsequent increase in the VEGF-induced release of von Willebrand factor causes the release of a multitude of proangiogenic factors from the activated platelets, such as VEGF, platelet-derived growth factor (PDGF), transforming growth factor, epidermal growth factor, and angiopoietin-1 (ANGPT-1) (21). The effect of this release in angiogenesis has been shown i*n vitro*, where activated platelets are able to induce tube formation of human umbilical vein endothelial cells in matrigel tube formation assays. This has been achieved through the secretion of endothelial stimulating factors, as well as by direct cellular interactions (22). In aortic ring assays, platelets and platelet releasates induced a marked increase in angiogenesis (23).

Another hint for the importance of platelets in the process of neoangiogenesis is that platelets are activated within the tumor vasculature, subsequently secreting their VEGF-rich releasate in the tumor tissue (24–26). Also, in animal models of breast cancer and renal cell cancer, it has been demonstrated that platelets tend to adhere with increased affinity to angiogenic vessels, mediated by the upregulation of CD24 on tumor cells, thus releasing their pro-angiogenic content in the tumor microenvironment (27). There is a dramatic increase in platelet activation markers, such as P-selectin, an adherent molecule, and angiogenesis markers in the platelets of cancer patients. For example, platelet lysate from breast cancer patients contains significantly higher levels of VEGF, ANGPT-1, and P-selectin, as compared to normal controls (28). In addition, intra-platelet levels of VEGF and PDGF are increased in colorectal cancer patients compared to those of healthy controls (28). Notably, these angiogenic regulators are detected in the early phase, and their levels are associated with clinical parameters (28–30). As has already been mentioned, VEGF plays the most important role in this process. This is supported in relevant studies, such as a meta-analysis by Kut et al., which demonstrated that, in cancer patients, the concentration of VEGF was 413 000 pg/ml, as compared to only 216 000 pg/ml in healthy controls (31). It has also been found that this pro-angiogenic factor may function as a diagnostic and prognostic biomarker, since it appears to predict cancer progression (32). Notably, although PDGF has also been associated with tumor growth and angiogenesis in many studies, it has not been attributed a specific role in cancer progression (33).

Additionally, platelets have been shown to recruit endothelial progenitor cells (EPCs) from bone marrow and increase their angiogenic potential. EPCs are also involved in neoangiogenesis (34). This has been demonstrated in B16-F10 tumors implanted in mice, an effect that has been attributed primarily to the platelet stromal-derived factor-1 (SDF-1) (35, 36), and secondarily to other factors, such as VEGF and ANGPT-1, which are also secreted by the activated platelets as described before (37, 38). Moreover, it seems that platelets contribute to the differentiation and maturation of EPCs that will eventually become the endothelial cells that line the novel blood vessels of the early metastatic niche (34).

Finally, platelets seem to have a role in the formation of the lymphatic network of the tumor as well, since a-granules contain VEGF-C, the most important regulator of lymphangiogenesis (39). Furthermore, a connection has been found between podoplanin, expressed in various cancer types, and TCIPA (40). Although it has not yet been confirmed that platelets do indeed guide the creation of lymphatic vessels in the tumor microenvironment, there is at least one study in esophageal cancer that has demonstrated an association between platelet counts, both in the circulation and in the tumor microenvironment, and lymphangiogenesis (41).

### Impairing the Host Defense Mechanisms

As previously mentioned, platelets protect CTCs from NK-mediated lysis during their voyage through the circulation. Although that may appear as a mostly mechanical process, it has been shown that platelets need to undergo activation in order to shield tumor cells from lysis (42). Platelet activation leads to the expression of the glucocorticoid-induced TNF-related ligand (GITRL), a member of the TNF receptor superfamily, on their surface membrane. GITRL binds to its receptor on the NK cell membrane and inhibits the cytotoxic properties of the latter, by impairing its lytic activity and the secretion of IFN-γ (43). It has been shown that the protective role of platelets toward tumor cells is not limited in the context of CTCs, but it is a phenomenon observed in the tumor microenvironment as well. It has been demonstrated that the releasate from activated platelets contains a multitude of soluble factors that exert an inhibitory effect on NK cells. For example, TGF-β secreted during TCIPA has been found to downregulate natural killer group 2D (NKG2D) immunoreceptor, impairing lytic activity and IFN-γ secretion (44). Also, it has been hypothesized that the transfer of platelet-derived MHC-I onto the surface of the tumor cells during aggregation, may further limit the NK-mediated attack of the immune system to the developing metastatic niche (45).

Platelets also interact with other immune cells, apart from NK, like macrophages and T-cells, and their effects on them may contribute in the creation of an immunosuppressive microenvironment. For example, the micrometastatic niche is rich in platelet-derived and tumor cell-derived TGF-β, which is suppressive for both CD4+ and CD8+ T-cell functions as well (46). Also, there is evidence that platelets represent the main source of functional TGF-β, both systemically and in the tumor microenvironment, through the expression of TGF-β docking receptor Glycoprotein A Repetitions Predominant (GARP). Thus, platelets constrain T-cell immunity through a GARP-TGF-β axis (47). Conclusively, platelets seem to have a substantial contribution in the induction of a localized state of dormancy in the host defense mechanisms, a situation that is vital for the development of the early metastatic foci.

Moreover, platelets have been implicated in the activation of neutrophils and the creation of neutrophil extracellular traps (NETs), in a process called NETosis (48). The interaction between platelets and neutrophils is bidirectional since, on one hand, platelet TLR4 triggers NETosis, and on the other, extracellular DNA from NETs triggers platelet activation (49). Also, this extracellular DNA contributes to cancer-associated thrombosis, which confers a dismal prognosis and represents the second-leading cause of death in cancer patients (47).

## Preclinical Evidence and Experimental Models

There exists enough evidence from preclinical studies and experimental models to support the pivotal role of platelets in the creation of the early metastatic niche (50, 51). Gasic et al. were the first to offer experimental evidence for the role of platelets in cancer. More specifically, in an experimental mouse model, it was demonstrated that the number of metastases in mice can be decreased by reducing the number of host platelets before tumor inoculation, and that this effect is independent of the method used to decrease the platelets (neuraminidase or anti-platelet serum) (52). This finding has been reproduced in other studies as well (53).

On the contrary, when injecting thrombocytopenic mice with human-derived platelets, the rate and extent of metastatic spread increased substantially (54). These experiments provide hints for the existence of a potential correlation between the absolute platelet count and cancer progression that can even be analyzed quantitatively.

Furthermore, Kerr et al., also workingon mouse models, demonstrated that platelets facilitate communication between pre-metastatic tumor cells and their pre-metastatic niche in bone tissue (10). Also, Labelle et al. showed that platelets induce the recruitment of granulocytes through the secretion of CXCL5 and CXCL7, promoting the creation of early metastatic sites (4). Finally, blocking of platelet–CTC interaction has also been evaluated as a method to reduce metastasis. Recently, Gareau et al. demonstrated that blocking this interaction using the P2Y12 inhibitor ticagrelor, reduced the number of metastasis and prolonged survival in a murine breast cancer model (55).

## Potential Clinical Relevance and Future Perspectives

Thrombocytosis of malignancy constitutes a well-known paraneoplastic syndrome, which is promoted by a multitude of cancer-related cytokines and growth factors, such as G-CSF, GM-CSF, IL-1, IL-6 and, more importantly, TPO (56–58). TPO is the main cytokine responsible for the stimulation of megakaryocyte production and platelet development, and has been found to be elevated in certain tumor types, such as in ovarian cancer (59, 60). Thrombocytosis in cancer patients is a common finding, and it is correlated with adverse prognosis. Several studies have reported that cancer incidence increases with increasing platelet count, and for those with an absolute platelet number more than 3.5 × 10^11^/L, the risk has been estimated to reach 3% in 1 year of observation (61, 62).

Based on the experimental data, targeting platelets appears a promising approach against cancer itself. Most of the clinical trials have evaluated aspirin, perhaps the most well-studied antiplatelet drug, as an anticancer agent. In a 2012 meta-analysis of 5 randomized clinical trials (RCT), aspirin has been found to reduce the risk of metastasis and the risk of death by cancer in patients with adenocarcinoma, irrespective of the organ of origin (63). In another meta-analysis of 15 RCTs, that included a substantial number of participants, daily aspirin was also found to reduce cancer deaths (63). However, other RCTs have found no such correlation (64, 65). Several clinical trials evaluating the effect of aspirin on cancer are currently ongoing^1^^,^^2^^,^^3^. One such large trial, the ADD-ASPIRIN Trial, is currently recruiting patients who previously had treatment for early cancer of the breast, stomach, esophagus, prostate and colon ^3^. The aim is to test whether 5 years of aspirin prophylaxis post initial treatment for cancer, can prevent or delay cancer recurrence. The hypothesis that aspirin exerts its anticancer actions by inhibiting the formation of the pre-metastatic niche has recently been tested in a murine experimental model for lung metastasis. It has been found that thromboxane A2 (TXA2) was the prostanoid product of COX-1 responsible for this anti-metastatic effect. Inhibition of the COX-1/TXA2 pathway in platelets decreased their aggregation on tumor cells, limited endothelial activation and the adhesion of tumor cells to the endothelium, and impaired the recruitment of metastasis-promoting monocytes/macrophages, thus diminishing the formation of pre-metastatic niche (66).

Previous studies had shown that other platelet activation pathways could contribute to the establishment of the intravascular metastatic niche. In particular, Clopidogrel a P2Y12 receptor antagonist, and eptifibatide an αIIbβ3 integrin inhibitor, two drugs used in the clinical practice to reduce platelet aggregation, were found to be associated with reduction in experimental metastasis (14, 67). This finding was not confirmed in the recent study by Lucotti et al. (66). However, further studies are required to explore the role of all possible platelet activation pathways at different stages of metastatic progression including the stages of epithelial—mesenchymal transition and extravasation (14, 68). This approach will potentially lead to the identification of new therapeutic targets and consequently antiplatelet agents to be used against the micrometastatic niche formation.

Finally, the creation of modified platelets that retained platelet binding functions but were incapable of functional activation and aggregation, termed “platelet decoys,” led to encouraging results in mouse models, where simultaneous injection of the platelet decoys with tumor cells inhibited metastatic tumor growth (69). The production of reversible drug-free antiplatelet agents by modifying human platelets, is of particular clinical importance as it carries the potential of stopping the formation of metastasis and the burden this is associated with in patients with cancer.

## Conclusion

Although the role of platelets in cancer progression is not limited to the preparation and maintenance of the metastatic microenvironment, this specific function is of utmost importance since the few early clusters of metastasizing tumor cells are extremely vulnerable and subject to many dangers in their surrounding environment. Accumulated data from preclinical studies and experimental models support the hypothesis that platelets contribute in every stage of the formation of the pre-metastatic niche. It is also possible that antiplatelet drugs, especially aspirin, exhibit at least part of their anticancer properties by impairing the formation of a suitable microenvironment for the development of metastases.

Whilst ongoing preclinical work is expected to shed light on additional platelet activation pathways at different stages of metastatic progression, several clinical trials aiming to evaluate antiplatelet agents in the treatment and prevention of cancer progression are currently ongoing.

## Author Contributions

SG and AC developed the idea and drafted the manuscript. SG created the figure. RJ critically reviewed the manuscript and contributed new ideas. The final manuscript was edited by AC and approved by all authors.

### Conflict of Interest

The authors declare that the research was conducted in the absence of any commercial or financial relationships that could be construed as a potential conflict of interest.

## Footnotes

^1^Aspirin for Dukes C and HighRisk Dukes B Colorectal Cancers (ASCOLT). ClinicalTrials.gov Identifier: NCT00565708.

^2^Finding the Best Dose of Aspirin to Prevent Lynch Syndrome Cancers (CaPP3 Israel). ClinicalTrials.gov Identifier: NCT02497820.

^3^ADD-ASPIRIN Trial website. http://www.addaspirintrial.org
